# COVID-19 vaccines in patients with decompensated cirrhosis: a retrospective cohort on safety data and risk factors associated with unvaccinated status

**DOI:** 10.1186/s40249-022-00982-0

**Published:** 2022-05-16

**Authors:** Zhujun Cao, Chenxi Zhang, Shuang Zhao, Zike Sheng, Xiaogang Xiang, Ruokun Li, Zhuping Qian, Yinling Wang, Bin Chen, Ziqiang Li, Yuhan Liu, Baoyan An, Huijuan Zhou, Wei Cai, Hui Wang, Honglian Gui, Haiguang Xin, Qing Xie

**Affiliations:** 1 Department of Infectious Diseases, Ruijin Hospital, Shanghai Jiao Tong University School of Medicine, Shanghai, 200025 China; 2 The First People’s Hospital of Yunnan Province, Medical School, Kunming University of Science and Technology, Kunming, China; 3 Department of Radiology, Ruijin Hospital, Shanghai Jiao Tong University School of Medicine, Shanghai, China; 4 Department of Nursing, Ruijin Hospital, Shanghai Jiao Tong University School of Medicine, Shanghai, China; 5 Department of Hepatology, The Affiliated Infectious Diseases Hospital of Soochow University, Suzhou, China; 6 Department of Infectious Diseases, Wuwei People’s Hospital, Gansu, China

**Keywords:** SARS-CoV-2, COVID-19, Vaccine, Decompensated cirrhosis, Acute-on-chronic liver failure, Liver transplantation

## Abstract

**Background:**

Safety data reported from the large-scale clinical trials of the coronavirus disease 2019 (COVID-19) vaccine are extremely limited in patients with decompensated cirrhosis. The vaccination campaign in this specific population could be difficult due to uncertainty about the adverse events following vaccination. We aimed to assessed the COVID-19 vaccination rate, factors associated with unvaccinated status, and the adverse events following vaccination in patients with decompensated cirrhosis.

**Methods:**

This is a retrospective study from Ruijin Hospial (Shanghai, China) on an ongoing prospective cohort designed for long-term survival analysis of decompensated cirrhotic patients who recovered from decompensating events or acute-on-chronic liver failure (ACLF) between 2016 and 2018. We assessed the COVID-19 vaccination rate, the number of doses, type of vaccine, safety data, patient-reported reasons for remaining unvaccinated, factors associated with unvaccinated status, and the adverse events of COVID-19 vaccine. Binary logistic regression was used for identifying factors associated with unvaccinated status.

**Results:**

A total of 229 patients with decompensated cirrhosis without previous SARS-CoV-2 infection participated (mean age, 56 ± 12.2 years, 75% male, 65% viral-related cirrhosis). Mode of decompensation were grade II‒III ascites (82.5%), gastroesophageal varices bleeding (7.9%), hepatic encephalopathy (7.9%). Eighty-five participants (37.1%) received at least one dose of vaccination (1 dose: *n* = 1, 2 doses: *n* = 65, 3 doses: *n* = 19) while 62.9% remained unvaccinated. Patient-reported reasons for remaining unvaccinated were mainly fear of adverse events (37.5%) and lack of positive advice from healthcare providers (52.1%). The experience of hepatic encephalopathy (*OR* = 5.61, 95% *CI*: 1.24–25.4) or ACLF (*OR* = 3.13, 95% *CI*: 1.12–8.69) and post-liver transplantation status (*OR* = 2.47, 95% *CI*: 1.06–5.76) were risk factors of remaining unvaccinated independent of residential areas. The safety analysis demonstrated that 75.3% had no adverse events, 23.6% had non-severe reactions (20% injection-site pain, 1.2% fatigue, 2.4% rash) and 1.2% had a severe event (development of acute decompensation requiring hospitalization).

**Conclusions:**

Patients with decompensated cirrhosis in eastern China are largely remained at unvaccinated status, particularly those with previous episodes of ACLF or hepatic encephalopathy and liver transplantation recipients. Vaccination against COVID-19 in this population is safe.

**Graphical Abstract:**

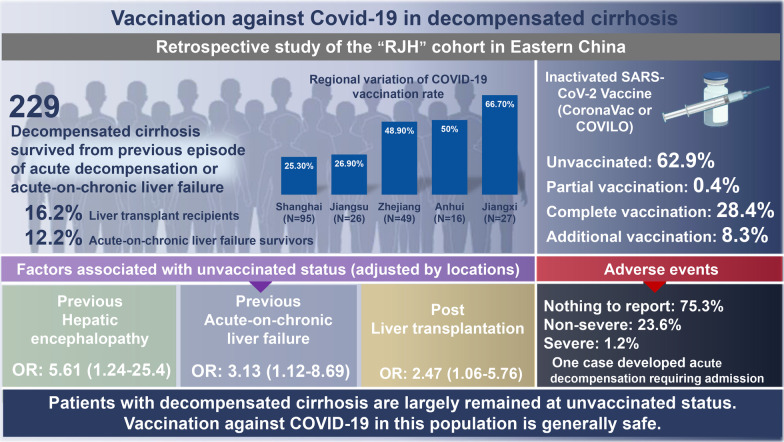

**Supplementary Information:**

The online version contains supplementary material available at 10.1186/s40249-022-00982-0.

## Background

Severe acute respiratory syndrome coronavirus 2 (SARS-CoV-2) infection is a high-risk comorbid condition in patients with immune-compromised population. Coronavirus disease 2019 (COVID-19) related mortality in patients with cancer patients is significantly increased despite the absence of symptoms [1, 2]. Twenty percent of these asymptomatic patients may suffer for a long time as evident by their failure to get seroconversion of the COVID-19 antibody [3].

Patients with cirrhosis are at risk of SARS-CoV-2 infection due to innate and humoral immune dysfunction [4] with an increased risk of hospitalization, intensive care unit admission, and death [5–8]. In a national COVID-19 cohort study across the United States, SARS-CoV-2 infection in patients with cirrhosis was associated with 2.38 times all-cause mortality hazard within 30 days [5]. Baseline decompensated cirrhosis and high organ failure scores are predictors of mortality in cirrhosis following SARS-CoV-2 infection [5, 7, 9].

Based on the cumulating evidence, it is now recommended by major hepatology societies that patients with cirrhosis, particularly those with decompensation should be vaccinated against SARS-CoV-2 [10, 11]. Although the vaccine immunogenicity in patients with cirrhosis is inferior to that of the general population [12], receipt of an mRNA-based COVID-19 vaccine in patients with cirrhosis is associated with a 66.8% reduction in SARS-CoV-2 infection after 28 days of the first dose [13] and vaccination significantly reduces mortality despite breakthrough infections [14]. Nevertheless, 40% of the patients with cirrhosis deferred COVID-19 vaccination in a United States Veterans Health setting despite a wide access to COVID-19 vaccines [15]. Among them, younger patients, current smokers, and living in rural areas were associated with vaccination hesitancy [15]. Although the generalizability of these findings in other settings of cirrhosis remains unknown, the reported 40% acceptance rate of COVID-19 vaccination urges more efforts to develop strategies to guide vaccine campaigns. Despite that the COVID-19 vaccinations are provided for free to the public in China where people have strong trust in central government and high acceptance rate [16], concerns about the safety and side-effects of the vaccines still significantly decrease the willingness to get vaccinated [17]. Currently, safety data reported from the large-scale clinical trials of the COVID-19 vaccine were still extremely limited in patients with cirrhosis, with less than 0.1% of more than 100,000 participants [18]. It is therefore important to investigate the SARS-CoV-2 vaccine coverage and safety profiles in patients with decompensated cirrhosis in real-world settings.

To assist in the development of vaccination strategies during the ongoing pandemic, we assessed the COVID-19 vaccination rate, factors associated with unvaccinated status, and the adverse events following vaccination in an ongoing prospective cohort previously designed for long-term survival analysis of cirrhotic patients who recovered from decompensating events or acute-on-chronic liver failure (ACLF).

## Methods

### Study design, participants, and follow-up

This retrospective study was performed in the Ruijin Hospital (RJH) cohort of patients with established cirrhosis who were non-selectively admitted for decompensation or ACLF between 2016 and 2018. This cohort has been utilized for the comparisons of the clinical utility of different ACLF criteria in the management of hospitalized cirrhosis [19]. All patients are enrolled in the Ruijin Hospital (academic hospital), Shanghai, China and were actively followed-up via hospital information system and/or phone call at scheduled time-point (28-day post-enrollment, 90-day post-enrollment, 180-day post-enrollment and every year thereafter). An extra follow-up was performed between 3 and 15 January 2022 via telephone for vital status, COVID-19 vaccination status including the number of doses, type of vaccine, safety data, and reasons for keeping unvaccinated. The study was monitored by a data management committee (QX, HGX) to improve the consistency and accuracy of data.

Inclusion criteria for the RJH cohort: Patients aged between 18 and 80 who were nonelectively admitted with cirrhosis for ascites, gastroesophageal varices bleeding, hepatic encephalopathy (HE), bacterial/fungal infection, and/or jaundice (total bilirubin ≥ 5 mg/dL). The diagnosis of cirrhosis was either biopsy-proven or based on the usual clinical, laboratory, endoscopic, and radiologic diagnostic criteria.

Exclusion criteria for the RJH cohort: (1) pregnancy or lactation, (2) thepresence of human immunodeficiency virus infection, (3) the presence of hepatocellular carcinoma irrespective of size, (4) other nonhepatic disseminated malignancies, (5) previous solid organ transplant, (6) treatment with immunosuppressive agents for diseases other than severe hepatitis, or (7) severe extrahepatic diseases with expecting poor short-term survival.

To account for potential delays in the launch of the local COVID-19 vaccination strategy from national vaccination campaign advocation at the end of March 2021, non-survivors before April 30, 2021 were further excluded, one month after the national vaccination campaign began in population aged between 18 and 80 years in China. Detailed information on vaccination campaigns in China is provided in Additional file 1.

### Outcomes

The primary outcome was the unvaccinated rate as of 15 January 2022.

Secondary outcomes included safety data regarding local (pain, redness, swelling, and lymphadenopathy) and systemic solicited adverse events (fever, chills, headache, fatigue, myalgia, arthralgia, nausea and vomiting, diarrhea, rash) for 7 days post-vaccination and unsolicited adverse events after vaccination till 15 January 2022. Definition of a serious adverse reaction is defined as any adverse event at any dose: results in death or is life-threatening or requires hospitalization or prolongs the existing hospitalization.

### Data collection

Demographics, comorbidities, etiology of cirrhosis, mode of decompensation, episode of bacterial infection, acute kidney injury (AKI), and ACLF during the initial hospitalization since enrollment were prospectively collected in the RJH study.

Information on the current residential province/ municipality, Shanghai vs other areas, rural vs urban areas were collected. Vaccination information includes vaccination status, vaccine type, number of doses, and adverse events following vaccination if vaccinated; reasons for remaining unvaccinated if unvaccinated.

Predefined type of vaccine in the current study was based on the authorized vaccines for emergency use in China as of 15 January 2022, including Inactivated SARS-CoV-2 Vaccine (CoronaVac from Sinovac, COVILO from Sinopharm/BIBP or Sinopharm/WIBP), Adenovirus-vectored vaccine (Convidecia from CanSinoBIO), and recombinant Novel Coronavirus Vaccine (Zhifei Longcom) [20].

### Statistical analysis

Patient characteristics were presented according to the type of data: Mean ± SD and median [interquartile range (IQR)] for normal and skewed distributed continuous variables, respectively; counts (percentages) for categorical variables. Comparisons between two groups were performed using Student’s test, Mann–Whitney U test, Chi-square, or Fisher exact test as appropriate. The proportion of patients who remained unvaccinated was calculated in the full analysis set and compared among different regions in China. Univariable and multivariable logistic regression was used to identify variables associated with unvaccinated status. The safety analysis was conducted in the population who received at least one dose of COVID-19 vaccination. Based on the previous reported vaccination rate in cirrhosis, we suppose the vaccination rate would be 80% in non-LT recipient and 50% in LT recipient by the time of our follow-up. The power of our study was 93.15% (β = 0.068) based on the current sample size (LT 37, non-LT 192), using two group proportion tests, with significance level α = 0.05. In all statistical analyses, a 2-tailed *P* < 0.05 was considered statistically significant. Data handling and analysis were performed with R 4.1.2 (http://www.r-project.org/).

## Results

### Study population

**Fig. 1:**
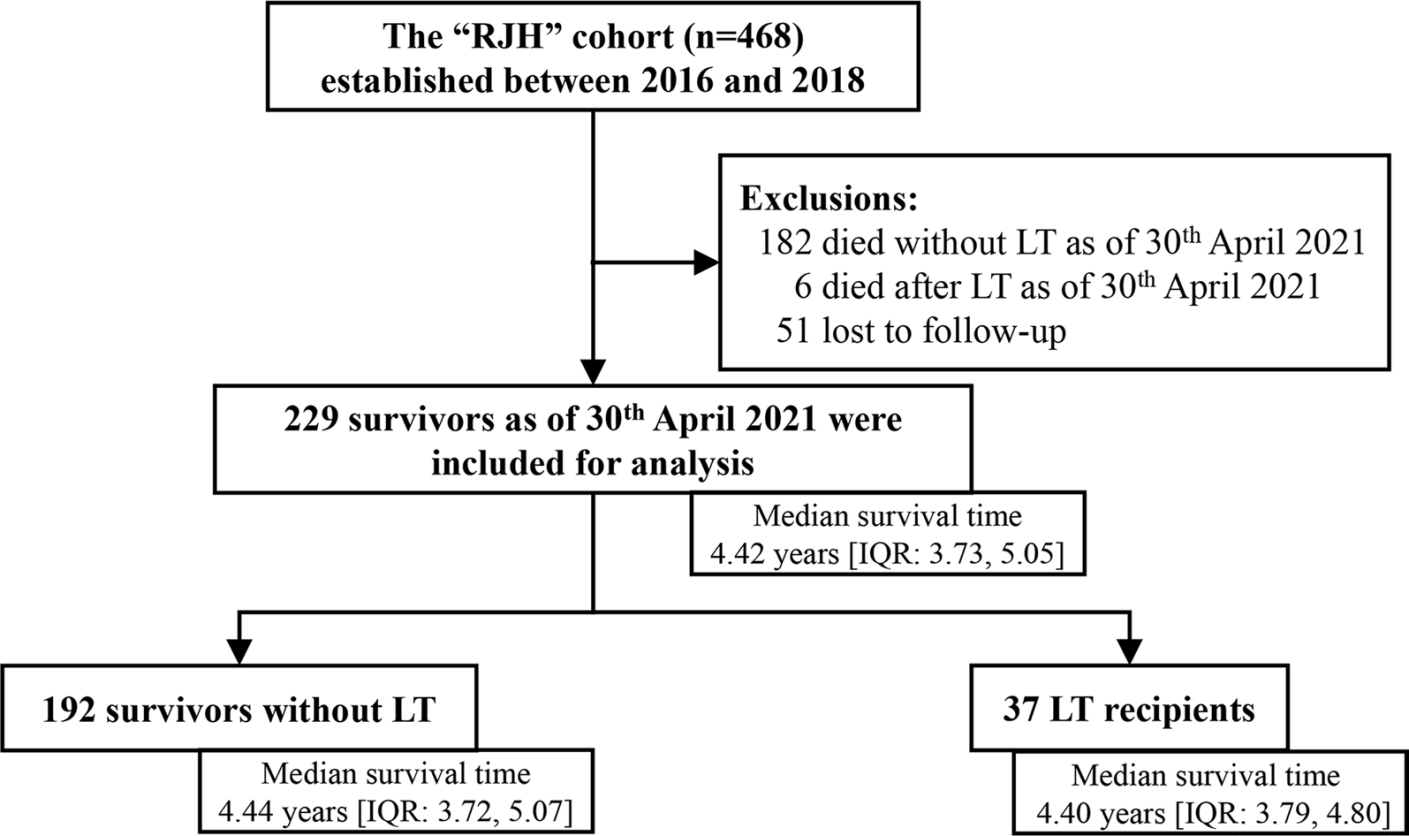
Patient flowchart. *RJH* Ruijin Hospital, *LT* liver transplant, *IQR* Interquartile range

Of all the participants in the RJH cohort, 51 were lost to follow-up and 188 patients died [182 of them (96.8%) did not receive an LT] as of 30 April 2021 (Fig. 1). The remaining 229 survivors [37 (16.2%) LT recipients and 192 (83.8%) survived without an LT] were included in the current study. The median survival time since enrollment in the RJH cohort was 4.42 years (IQR: 3.73, 5.05).

### Subject characteristics

Characteristics of patients included in this study is shown in Table 1 and compared between those with and without LT. These were mainly male patients (75.1%) with viral-related decompensated cirrhosis (65%), living currently in Shanghai and peripheral cities with a mean age of 56 ± 12.2 years. None of the participant in the current study was infected by SARS-CoV-2 by the time they were enrolled in the current study.

**Table 1:** Patient characteristics

Variables	All (*n* = 229)	Without LT (*n* = 192)	LT recipients (*n* = 37)	*P*-value
Male, (%)	172 (75.1)	142 (74.0)	30 (81.1)	0.48
Age (years), mean (*SD*)	56.00 (12.15)	56.51 (12.54)	53.35 (9.61)	0.15
Systemic hypertension, (%)	40 (17.5)	32 (16.7)	8 (21.6)	0.62
Type II Diabetes, (%)	34 (14.8)	27 (14.1)	7 (18.9)	0.61
Etiology of cirrhosis, (%)				0.49
Viral	149 (65.1)	121 (63.0)	28 (75.7)	
Alcohol	17 (7.4)	16 (8.3)	1 (2.7)	
AIH	13 (5.7)	10 (5.2)	3 (8.1)	
Cholestasis	10 (4.4)	9 (4.7)	1 (2.7)	
Others	22 (9.6)	19 (9.9)	3 (8.1)	
Multi	18 (7.9)	17 (8.9)	1 (2.7)	
Experience of acute deterioration, (%)				
Ascites (Grade II-III)	189 (82.5)	157 (81.8)	32 (86.5)	0.65
GEVB	18 (7.9)	13 (6.8)	5 (13.5)	0.29
HE	18 (7.9)	15 (7.8)	3 (8.1)	1.00
Bacterial/fungal infection	46 (20.1)	35 (18.2)	11 (29.7)	0.17
AKI	19 (8.3)	11 (5.7)	8 (21.6)	< 0.01
ACLF	28 (12.2)	15 (7.8)	13 (35.1)	< 0.001
Current living area (%)				
Rural	95 (41.7)	83 (43.2)	12 (33.3)	0.36
Location				0.99
Shanghai	95 (41.5)	79 (41.1)	16 (43.2)	
Zhejiang	49 (21.4)	42 (21.9)	7 (18.9)	
Jiangsu	26 (11.4)	21 (10.9)	5 (13.5)	
Jiangxi	27 (11.8)	23 (12.0)	4 (10.8)	
Anhui	16 (7.0)	14 (7.3)	2 (5.4)	
Others	16 (7.0)	13 (6.8)	3 (8.1)	

Values are number (%) for categorical variables and mean (*SD*) for Age

*AIH* auto-immune hepatitis, *GEVB* gastro-esophageal varices bleeding, *HE* hepatic encephalopathy, *AKI* acute kidney injury, *ACLF* acute-on-chronic liver failure, *LT* liver transplant, *SD* Standard deviation

No differences in age, gender, etiology of cirrhosis, comorbidities, and current living areas were observed between patients with and without LT. Mode of decompensation included moderate-to-large ascites, HE, GEVB. Other complications at the initial enrolment include bacterial/fungal infection, the rates of which were similar between the two groups, but there were significant more cases of AKI (21.6% vs 5.7%, *P* < 0.01) and ACLF (35.1% vs 7.8%, *P* < 0.01) amongst LT recipients.

### Vaccination status of the inactivated SARS-CoV-2 vaccine

**Fig. 2:**
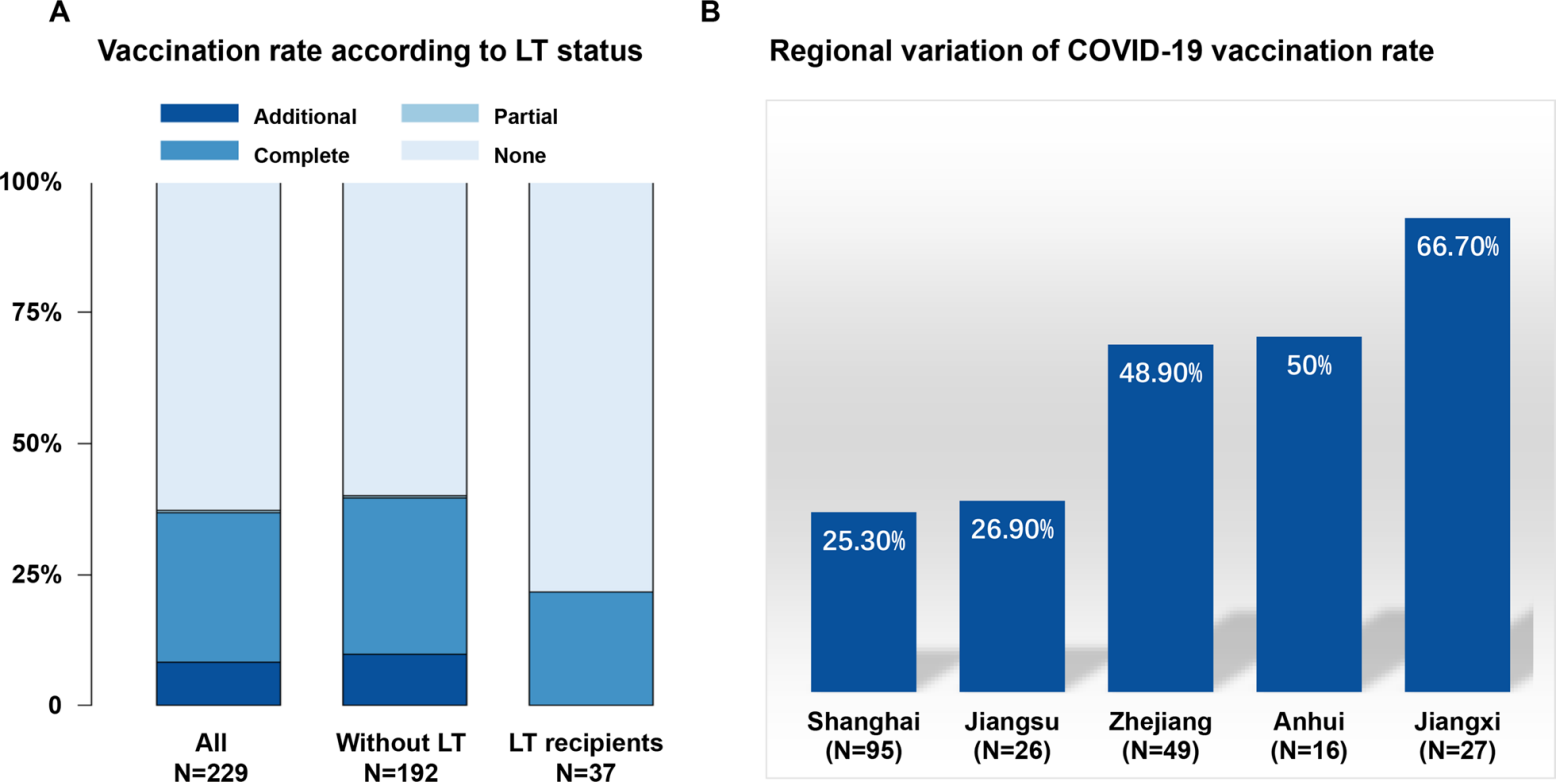
Vaccination rate according to LT status and residence area. Vaccination status was classified as none (0 dose), partial (1 dose), complete (2 doses) and additional (3 doses) and were calculated in all participant, without LT and LT recipients (**A**). Regional variation of vaccination rate was observed among different residence areas being lowest in Shanghai city and highest in Jiangxi province (**B**). *LT* liver transplant

As of 15 January 2022, 144 (62.9%) participants remained unvaccinated. A total of 85 (37.1%) participants received at least one dose of the COVID-19 vaccine (1 dose: *n* = 1 or 0.4%, 2 doses: *n* = 65 or 28.4%, and 3 doses: *n* = 19 or 8.3%) (Fig. 2A). All vaccination occurred when the participants were in conditons of stabilized decompensated cirrhosis. There is a significant variation in vaccination rates among different provinces/municipalities, from 25.3% in Shanghai to 66.7% in Jiangxi (Fig. 2B).

No statistically different distribution of vaccination status was observed between groups, but LT recipients were more likely to remain unvaccinated than those without LT (78.4% vs 59.9%, *P = *0.052), and no LT recipient received additional vaccination.

Among all the 85 vaccinated participants, 60 reported the type of vaccines, all of which were inactivated SARS-CoV-2 vaccines (CoronaVac = 47, COVILO = 11, Mixed = 2). There was no significant difference in the types of vaccine between groups.

### Patient-reported reasons and objective factors associated with unvaccinated status

**Fig. 3:**
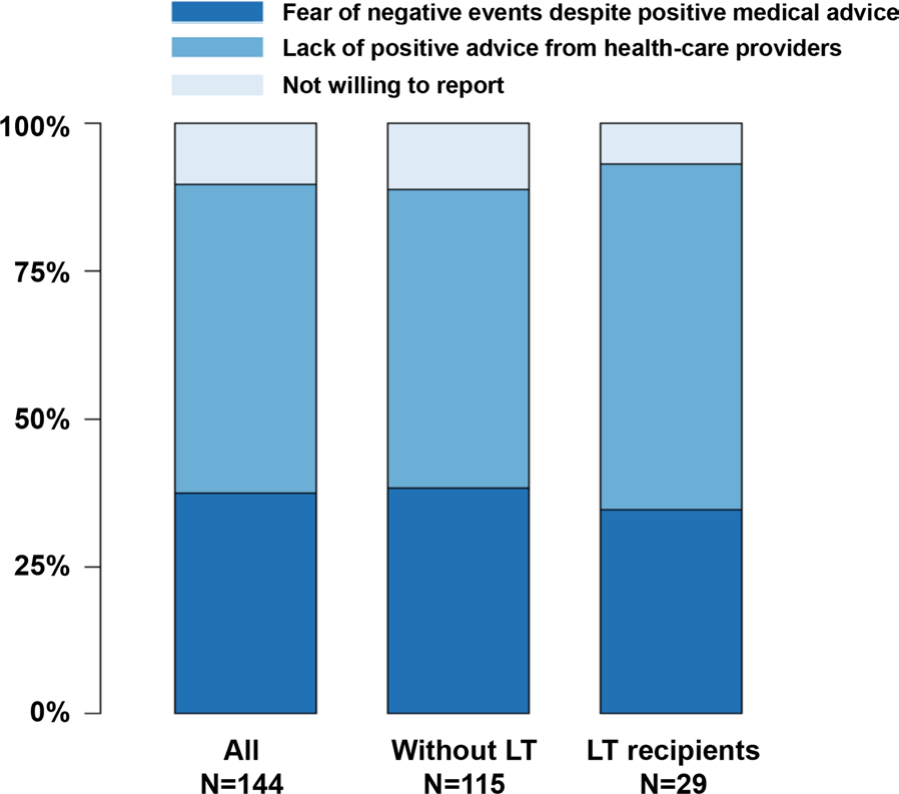
Patient-reported reasons for remaining unvaccinated against COVID-19. LT: Liver transplant

Among the 144 participants who remained unvaccinated, 75 (52.1%) were not vaccinated for COVID-19 due to the lack of positive medical advice, 54 (37.5%) had fear of negative side events despite positive medical advice, and 15 (10.4%) were unwilling to report reasons (Fig. 3). The reported reasons for vaccination hesitancy were not different between those with and without LT.

Comparing to vaccinated patients, those remained unvaccinated were more likely to have experience of HE (*OR* = 5.19, 95% *CI*: 1.16–23.15, P = 0.03) or ACLF (*OR* = 3.04, 95% *CI*: 1.11–8.33, P = 0.03), LT recipients (*OR* = 2.43, 95% *CI*: 1.05–5.59, P = 0.04), or currently living in Shanghai city (*OR* = 2.47, 95% *CI*: 1.39–4.39, P < 0.01) (Table 2).

**Table 2:** Factors associated with unvaccinated status for COVID-19

Variables	Unvaccinated (*n* = 144)	Vaccinated (*n* = 85)	*P*-value	Univariate analysis		Multivariate analyses	
				Odds ratio (95% *CI*)	*P*-value	Adjusted odds ratio (95% *CI*)	*P*-value
Male, (%)	110 (76.4)	62 (72.9)	0.67	1.20 (0.65–2.22)	0.56		
Age (years), mean (*SD*)	57.4 (11.8)	56.3 (12.8)	0.52	1.01 (0.99–1.03)	0.52		
Systemic hypertension, (%)	27 (18.8)	13 (15.3)	0.63	1.28 (0.62–2.64)	0.51		
Type II Diabetes, (%)	26 (18.1)	8 (9.4)	0.11	2.12 (0.91–4.93)	0.08		
Etiology of cirrhosis, (%)							
Viral	96 (66.7)	53 (62.4)	0.30	1.44 (7.08–0.29)	0.83		
Alcohol	13 (9.0)	4 (4.7)					
AIH	9 (6.2)	4 (4.7)					
Cholestasis	7 (4.9)	3 (3.5)					
Others	10 (6.9)	12 (14.1)					
Multi	9 (6.2)	9 (10.6)					
Experience of acute deterioration, (%)							
Ascites (Grade II–III)	119 (82.6)	70 (82.4)	1.00	1.02 (0.50–2.06)	0.96		
GEVB	14 (9.7)	4 (4.7)	0.27	2.18 (0.69–6.86)	0.18		
HE^a^	16 (11.1)	2 (2.4)	0.03	5.19 (1.16–23.15)	0.03	5.61 (1.24–25.4)	0.025
Bacterial/fungal infection	31 (21.5)	15 (17.6)	0.60	1.28 (0.65–2.54)	0.48		
AKI	13 (9.0)	6 (7.1)	0.78	1.31 (0.48–3.58)	0.60		
ACLF^a^	23 (16.0)	5 (5.9)	0.04	3.04 (1.11–8.33)	0.03	3.13 (1.12–8.69)	0.029
LT recipient (%)^a^	29 (20.1)	8 (9.4)	0.05	2.43 (1.05–5.59)	0.04	2.47 (1.06–5.76)	0.037
Current living area (%)							
Rural	54 (37.8)	41 (48.2)	0.16	0.65 (0.38–1.12)	0.12		
Shanghai^b^	71 (49.3)	24 (28.2)	< 0.01	2.47 (1.39–4.39)	< 0.01	2.55 (1.41–4.59)	0.002

Values are number (%) for categorical variables and mean (*SD*) for continuous variables

Odds ratio was determined by the logistic regression analysis taking “Unvaccinated status” as outcome

^a^Odds ratio was adjusted by residence in Shanghai in the multivariate analyses

^b^Odds ratio was adjusted by the experience of HE, ACLF and LT in the multivariate analyses

*AIH* auto-immune hepatitis, *GEVB* gastro-esophageal varices bleeding, *HE* hepatic encephalopathy, *AKI* acute kidney injury, *ACLF* acute-on-chronic liver failure, *LT* liver transplant, *SD*: Standard deviation

Multivariate logistic regression analysis adjusting for residence in Shanghai demonstrated that HE experience (adjusted *OR* = 5.61, 95% *CI*: 1.24–25.4, *P* = 0.03), ACLF experience (adjusted *OR* = 3.13, 95% *CI*: 1.12–8.69, *P *= 0.03) and LT (adjusted *OR* = 2.47, 95% *CI*: 1.06–5.76, *P* = 0.04) recipient status remained statistically significant risk factors of unvaccinated status (Table 2).

### Safety analysis

Safety analysis was performed on 85 participants who received at least one dose of the SARS-CoV2 vaccine. Sixty-four (75.3%) patients did not report side events after SARS-CoV-2 vaccination and the remaining 21 (24.7%) participants reported at least one side event (Table 3). Overall, adverse events were mostly non-severe with injection-site pain (20%) being the most common one. Systemic side events were reported by 1 patient with fatigue and 2 patients with rash. All the systemic symptoms were transient and recovered without medication. Severe adverse event was reported by 1 patient for the acute development of large ascites after the first dose of COVID-19 vaccination. He recovered after hospitalization, and deferred receiving the second dose of the vaccine. No patient reported thromboembolic events or myocarditis.

**Table 3:** Summary of adverse events following COVID-19 vaccination in patients with decompensated cirrhosis

Adverse events following immunization	All (*n* = 85)	Without LT (*n* = 77)	LT recipients (*n* = 8)	*P* value
None	64 (75.3)	58 (75.3)	6 (75.0)	1.0
Severe				
Acute decompensation requiring admission	1 (1.2)	1 (1.3)	0 (0.0)	1.0
Non-severe				
Local				
Injection-site pain	17 (20.0)	15 (19.5)	2 (25.0)	0.66
Systemic				
Fatigue	1 (1.2)	1 (1.3)	0 (0.0)	1.0
Rash	2 (2.4)	2 (2.6)	0 (0.0)	1.0

Values are number (%)

## Discussion

This study demonstrated that, in the RJH cohort, which consists of patients from the eastern provinces of China, more than half of the patients with decompensated cirrhosis who survived from previous episodes of decompensation or ACLF remained unvaccinated against COVID-19 despite the ongoing pandemic. Lack of positive advice from the medical providers and fear of negative events from COVID-19 vaccination were the main reasons for remaining unvaccinated. Vaccination rate varied among different regions and experience of HE, ACLF or LT were identified as risk factors of unvaccinated status independent of living area. Among all the vaccinated patients, side events were reported in one-quarter, mainly with injection-site pain.

Patients with cirrhosis, particularly those at decompensated stage should be prioritized for COVID-19 vaccines as recommended by the major liver societies [10, 11]. However, the vaccination rate in cirrhosis is relatively low (~ 60%) in a report of the Veterans Health Administration data across the United States [13, 15]. The vaccination rate further decreased in decompensated cirrhosis (37.1%) as revealed by our current study, sharply contrasting to the overall 90% rate of COVID-19 vaccination in the general population in our country (China) as of 17 Jan 2022 [21]. That is, despite wide access to free vaccines for general public, specific population like decompensated cirrhosis, LT recipients are lag far behind. Although it is unclear whether this phenomenon also exists in other regions, more efforts are needed to further advocate the necessity of vaccination by the hepatology community.

Several cases developed acute liver injury following COVID-19 vaccination [22], including acute exacerbation of AIH [23, 24]. Such acute insult of the liver could act as a trigger of ACLF in decompensated cirrhosis. Therefore, the uncertainties of safety contribute mostly to the low vaccination rate in our cohort. The lack of published safety data from COVID-19 vaccination in this particular population makes it difficult for the patient to decide and also makes the healthcare provider hard to advocate with evidence. Ninety percent of the patients remained unvaccinated either because of lacking positive medical advices or fear of side events despite positive medical advice. With the analysis of the safety dataset, we demonstrate that in decompensated cirrhosis, COVID-19 vaccination is generally safe, similar to the previous reports in chronic liver disease [25], non-alcoholic fatty liver disease [26], LT recipients, cirrhosis (mainly compensated stage) [9] and chronic hepatitis B [27]. Patients with decompensated cirrhosis are especially vulnerable to develop severe COVID-19 due to immune dysfunction [5, 9, 28–30]. The benefits of COVID-19 vaccination greatly surpass the risks of the post-vaccination adverse events. We, therefore, recommend hepatologists or physicians to include discussions about COVID-19 vaccination with decompensated cirrhotic patients who remained unvaccinated and educate them on the risks and benefits while correcting their attitude against vaccination.

Identification of risk factors associated with unvaccinated status is critical to target interventions. In our cohort, there is a significant variation of vaccination rates among different provinces/municipalities, being the lowest in Shanghai (25.3%) and highest in Jiangxi (66.7%). This could partly be explained by the exposure risk analysis showing that Shanghai had the lowest number of domestically transmitted COVID-19 cases among all the investigated regions (Additional file 1: Fig. S1). Of note, geographical variation of the COVID-19 vaccine coverage in a specific population is also subject to regional policy, vaccine accessibility, delivery strategy, individual factors, etc. [31–33]. Taking advantage of the pre-collected information on the acute episode of GEVB, moderate-to-large ascites, HE, AKI, Infection or ACLF in our database, we demonstrated for the first time that, decompensated cirrhosis with experience of ACLF, HE or LT were more likely to defer COVID-19 vaccination. These underlying conditions remained statistically significant after adjusting for the location of residence in Shanghai. This information is of great importance to target potential unvaccinated individuals in patients with decompensated cirrhosis, particularly in regions where healthcare facilities are overwhelmed with COVID-19 patients. It has been reported that immunogenicity of inactivated SARS-CoV-2 Vaccine (BBIBP-CorV) are compromised in patients with underlying disease such as cancer patients [3, 34]. In patients with decompensated cirrhosis, approximately one-third of the cirrhotic patients had low cellular vaccine response [9] requiring additional primary shot and booster shot to enhance immunogenicity. It would be too late for patients with decompensated cirrhosis to start primary shots when the COVID-19 invades into their living communities.

Strengths of the present study include a large cohort of patients with a well-documented history of decompensating events and more importantly, ACLF episodes. It is currently the first and largest study to describe vaccination acceptance and safety profile in decompensated cirrhosis who were poorly represented in the previous clinical trials of COVID-19 vaccines.

Limitations of the present study include the retrospective design, lacking information on the current disease severity, social economics, and other potential residual confounding factors associated with unvaccinated status. Patients included for analysis in the current study were those with decompensated cirrhosis who survived for more than 3 years from the previous decompensation, some of them might have been re-compensated, though it is hard to define this status. Secondly, serologic response data were not able to be captured since the patients had no regular serologic assessment after vaccination in China. Thirdly, cox regression would be more appropriate to investigate the factors associating with time-to-unvaccination, but is not available in our database because we did not collect the exact accessiable date for vaccination and the vaccinated date. The odds ratio used in our study overestimated the risk in our study due to the relative high rate of unvaccinated patients. Finally, the study was performed in a single-center setting in China during a period when the COVID-19 situation is under control. There is selection bias and it remains unclear whether our observations could be extrapolated to other settings.

## Conclusions

COVID-19 vaccination is generally safe but the acceptance rate was currently low in a cohort of decompensated cirrhosis who recovered from previous decompensation or ACLF. Urgent efforts from international and local hepatology societies, regional policymakers, vaccine delivery systems, and healthcare providers should be devoted to addressing the vaccination gap in this specific population. Previous episodes of HE, ACLF, or current post-LT status could help to predict unvaccinated status independent of region difference. More studies are needed to establish strategies for increasing vaccination coverage in decompensated cirrhosis to cope with the ongoing pandemic.

## Supplementary Information

**Additional file 1.** List of abbreviations, the vaccination campaign in China and the supplementary Figure 1 for the total confirmed COVID-19 cases as of 7 Feb 2022 in the five major areas involved in the current study.

## Data Availability

The data will be available upon requested.

## Abbreviations

SARS-CoV-2: Severe acute respiratory syndrome coronavirus 2
COVID-19: Coronavirus Disease disease 2019
ACLF: Acute-on-chronic liver failure
RJH: Ruijin Hospital
LT: Liver transplantation
GEVB: Gastro-esophageal varices bleeding
HE: Hepatic encephalopathy
AKI: Acute kidney injury
IQR: Interquartile range
