# Supplementary material for: COVID-19 vaccines in patients with decompensated cirrhosis: a retrospective cohort on safety data and risk factors associated with unvaccinated status
Source: Infect Dis Poverty. 2022 May 16;11:56. doi: 10.1186/s40249-022-00982-0 (PMC9108345; doi:10.1186/s40249-022-00982-0)

**SUPPLEMENTARY APPENDIX**

**List of abbreviations used in the main text**

SARS-CoV-2, Severe acute respiratory syndrome coronavirus 2

Covid-19, Coronavirus disease 2019

ACLF, Acute-on-chronic liver failure

RJH, Ruijin Hospital

LT, Liver transplantation

GEVB, Gastro-esophageal varices bleeding

HE, Hepatic encephalopathy

AKI, Acute kidney injury

IQR, Interquartile range

**The vaccination campaign in China**

The Covid-19 vaccination campaign in China began at the end of December 2021 and was first administered to high-risk groups, including those engaged in handling imported cold-chain products and people working in exposed sectors such as port inspection and quarantine, ship pilotage, aviation, fresh market, public transport, frontline healthcare providers and disease prevention and control staffs.

The campaign was extended to the general public aged ≥18 years since the first edition of the Guidance of SARS-CoV-2 Vaccination was released by the National Health Commission of the People's Republic of China on 29 March 2021 ([1](#_ENREF_1)).

**Reference**

1. National Health Commission of the People's Republic of China. Guidance of SARS-CoV-2 456 Vaccination (First version). Chinese Journal of Clinical Infectious Diseases, 2021, 14(02):89-90.

**Supplementary Figure 1**

**Total confirmed COVID-19 cases as of 7 Feb 2022 in the five major areas involved in the current study.**

Data were accessed on 7 Feb 2022 at: https://ncov.dxy.cn/ncovh5/view/pneumonia


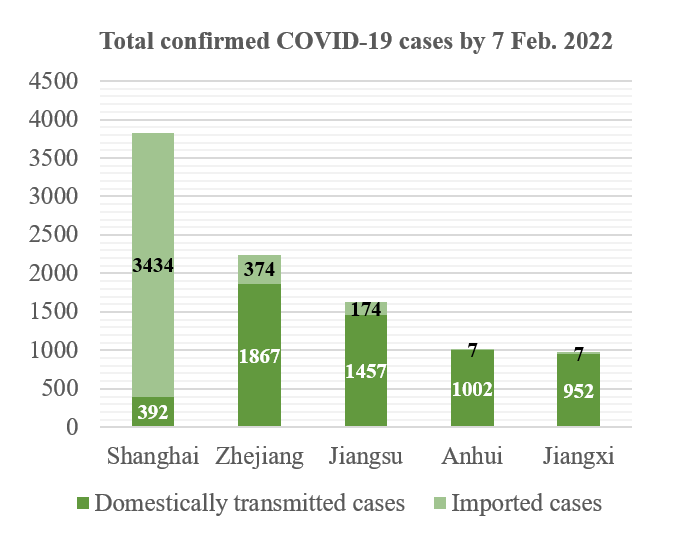

Supplement: Supplementary file 1 — Additional file 1. List of abbreviations, the vaccination campaign in China and the supplementary Figure 1 for the total confirmed COVID-19 cases as of 7 Feb 2022 in the five major areas involved in the current study. [file 40249_2022_982_MOESM1_ESM.docx]
